# Nonsurgical Treatment of a Massive Substance Loss

**DOI:** 10.1155/2013/716549

**Published:** 2013-08-29

**Authors:** Pasquale Fino, Fioramonti Paolo, Diego Massera, Vittoria Amorosi, Maria Giuseppina Onesti

**Affiliations:** Department of Plastic, Reconstructive and Aesthetic Surgery, University of Rome “Sapienza”, Policlinico Umberto I, Viale del Policlinico 155, 00161 Rome, Italy

## Abstract

Traumatic wounds are caused by severe trauma, resulting in lesions with extensive skin and subcutaneous tissue loss and damage to tissue viability. A “difficult wound” is a solution of continuity that does not heal spontaneously within three months. The factors that determine it may be as follows: a massive loss of substance, an infection, the presence of foreign bodies, or the clinical condition of the patient. We report a case of a 25-year-old man that presents a skin lesion on the anterior region of the left arm with extensive necrosis of skin and subcutaneous plants that involve the underlying muscle planes, caused by a trauma due to a car accident. In most of the lesions of such size and position, there is always a need for surgery. But in this case, considering the young age and the regenerative capacity of the patient, a quick and targeted antibiotic therapy was chosen, combined with debridement and worked with collagenase ointment.

## 1. Introduction

The increasing longevity and prevalence of trauma victims in hospitals, raising the frequency of so-called “difficult” wounds, attracted not only doctors and nurses but also health care administrators attention, concerned about the impact of costs of treating these conditions. In hospital, the care of these patients is generally associated with prolonged hospitalization time, use of expensive antibiotics, and need for daily dressings, with employment of a large team of specialized professionals [1–3].

A complex wound is difficult to heal when associated with one or more of the following risk factors: extensive skin loss, aggressive infection, impaired tissue viability (presence of ischemia and/or necrosis), and association with systemic diseases that compromise the normal processes of healing (diabetes, vascular disorders, vasculitis) [2, 3].

Traumatic wounds are caused by severe trauma, resulting in lesions with extensive skin loss and damage to the tissue viability, as detaching injuries in lower limbs, amputation of limbs and fingers, as well as bruises, lacerations and large crushes, with exposition of noble tissue [4, 5].

The following case represents a typical case of trauma with massive loss of substance infected and treated only with antibiotics and collagenase ointment.

## 2. Case Report

**Figure 1:**
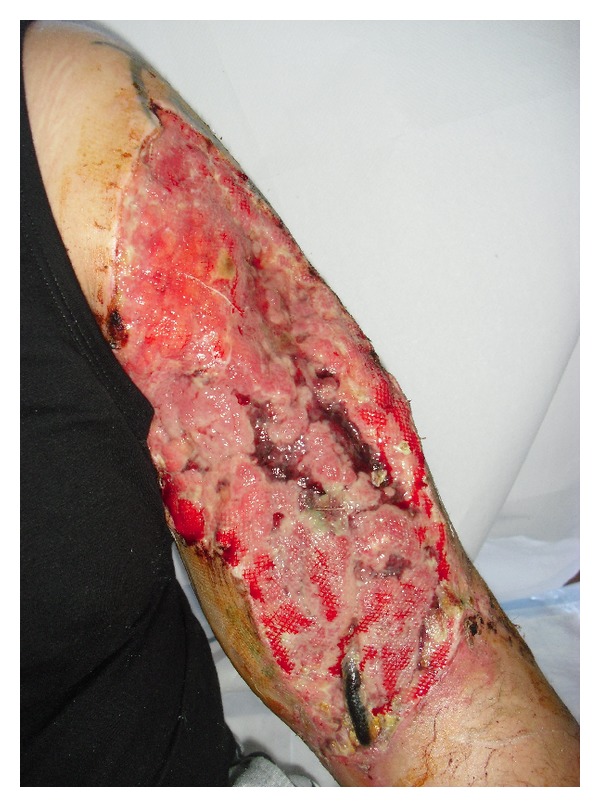
Preoperative appearance of the ulcerative skin lesion on the anterior region of the left arm with extensive necrosis of skin and subcutaneous plants involving the underlying muscle planes.

A 25-year-old man presented to our facility of difficult wounds one month after the accident with a skin lesion on the anterior region of the left arm with extensive necrosis of skin and subcutaneous plants involving the underlying muscle planes, caused by a trauma due to a car accident (Figure 1).

He was previously evaluated by the emergency, orthopedics, and vascular surgery departments of his city. The reports referred lacerated-contused with multiple massive loss of substance, probably torn tendon and vascular damage. The main lesion was clearly visible; it covered the entire anterolateral left arm and was infected.

The wound size was 40 × 15 cm; it had a sanious, fibrinous, secreting, and smelly bottom. A buffer was performed; it resulted positive for Pseudomonas Aeruginosa, and then an antibiotic therapy was prescribed based on fluoroquinolone cps. 500 mg, 2 cp./die for two weeks. 

The patient was treated at our facility of difficult wounds, Department of Plastic, Reconstructive, and Aesthetic Surgery in Rome over a period of three months. In this period, we performed daily dressings before debridement of the lesion through a collagenase plus hyaluronic acid ointment: Bionect Start (Fidia Pharmaceutical, Abano Terme, Italy). Each dressing consisted of four phases: disinfection with sodium hypochlorite 0.05% (Amukine Med 0,05%, Amuchina S.p.A., Genova) and povidone-iodine solution 10% (Betadine, Meda Pharma S.p.A, Milano), cleansing with saline solution, applying a layer of 2 mm ointment, and cover with a premedicated patch. After the first week, a considerable reduction of exudate, fibrin, pain, and smell was observed. An initial onset of granulation was already evident.

**Figure 2:**
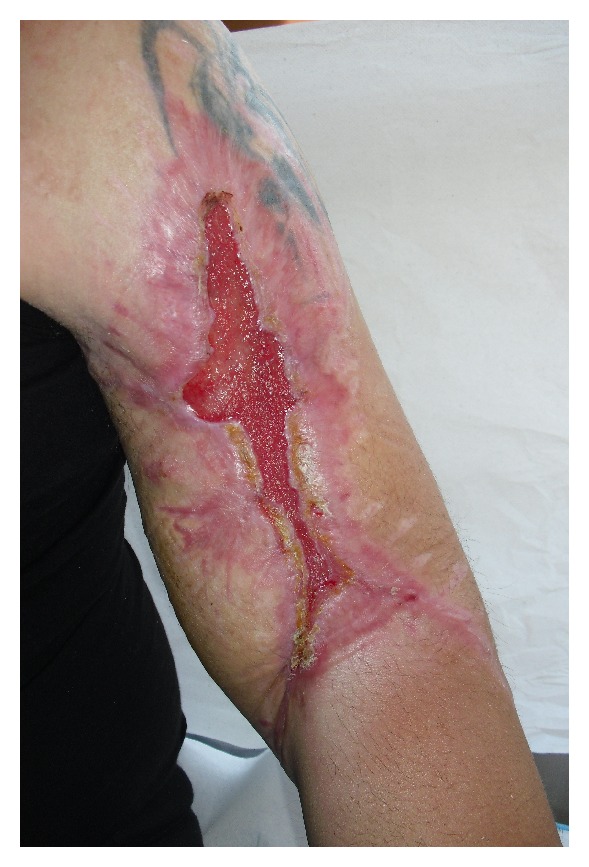
Granulation with reduction of the depth of the wound after two month.

After two months of dressings, we obtained the increase of the granulation with reduction of wound's depth from the bottom and the start of reepithelialization from the margins (Figure 2).

**Figure 3:**
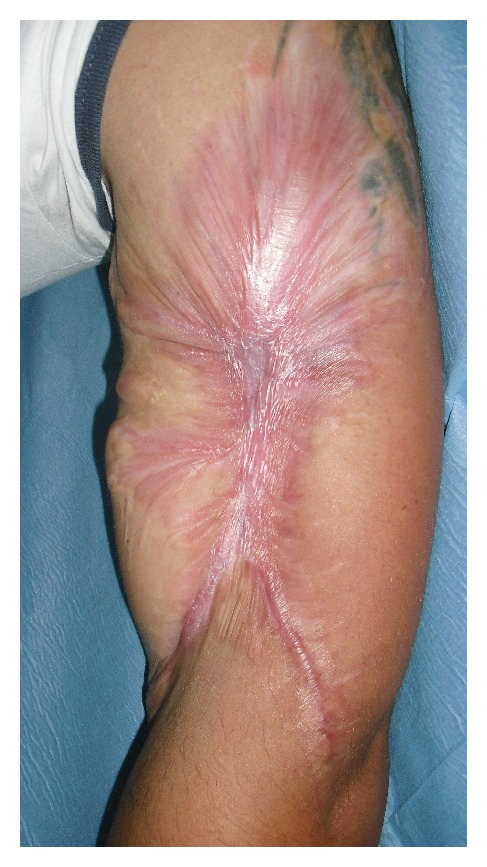
Complete reepithelialization of the wound after three months.

After three months, we obtained a complete healing (Figure 3).

## 3. Discussion

In most of the lesions of such size and position, the need for surgery is always necessary. But in this case, considering the young age and the regenerative capacity of the patient, what was necessary was a quick and targeted antibiotic therapy, combined with debridement and worked with collagenase ointment.

We obtained the closure of the wound in a relatively short period, considering the size, the type of lesion, and the infection, without resorting to surgery but using dressings as a new collagenase. The action of this ointment is to operate a chemical debridement of the wound bed through the action of the fibrinolytic enzymes (collagenase), maintaining the moist environment and giving protection in order to encourage granulation and the increase of the number of new cells called fibroblasts thanks to the presence of hyaluronic acid in the ointment.

It is a topical cream containing hyaluronic acid, bacterial fermented sodium hyaluron (0,2% w/w) salt, and bacterial collagenase obtained from nonpathogenic Vibrio alginolyticus (>2,0 nkat1/g). The use of collagenase is based on performing lysis of fibrin and necrotic tissue. The topical administration of collagenase increases the effect of macrophagic collagenase, responsible for wound debridement by splitting and breaking down proteins which hold eschar (dead and devitalised material) on the wound. This collagenase also contains hyaluronic acid (HA) that above all generates a microenvironment stimulating the secretion of growth factors and proliferation and migration of fibroblasts, endothelial cells, keratinocytes, and angiogenesis and has a positive effect on the inflammatory response. Moreover, HA is also capable of regulating the water balance acting on osmotic pressure and flow resistance and selectively sieving the diffusion of plasma and matrix proteins [6–9].

## 4. Conclusion

The therapeutic choice was rewarded relatively quickly, with a complete resolution of the wound through a noninvasive technique. Avoiding hospitalization achieved a reduction of risks for of the patient and a reduction costs for the NHS (National Health Service). For the residual scar, we plan on performing lipofilling to restore the volume and improve the trophism and the quality of the scar tissue. Then, if the result is not satisfactory we may also perform surgical revision of residual scars with a debridement of the scarring bridles.

The Bionect Start as well as allowing the healing of the wound also decreased significantly the pain felt by the patient, the amount of exudate, and the bad smell improving in a nonnegligible way his quality of life. In conclusion, we can say that the option of chemical debridement, when possible, is certainly better because of the risks, costs, and quality of life of patients. 

## References

[1] Morris JP, Wood WC, Chery GW, Hughes MA, Leaper DJ, Ferguson MWJ, Morris PJ, Wood WC (2001). Wound healing. *Oxford Textbook of Surgery*.

[2] Ferreira MC, Tuma P, Carvalho VF, Kamamoto F (2006). Complex wounds. *Clinics*.

[3] Harding KG, Morris HL, Patel GK (2002). Science, medicine, and the future: healing chronic wounds. *British Medical Journal*.

[4] Lee CK, Hansen SL (2009). Management of acute wounds. *Surgical Clinics of North America*.

[5] Schlatterer D, Hirshorn K (2008). Negative pressure wound therapy with reticulated open cell foam-adjunctive treatment in the management of traumatic wounds of the leg: a review of the literature. *Journal of Orthopaedic Trauma*.

[6] Lambert F, Couturaud B, Arnaud E, Champeau F, Revol M, Servant JM (2003). *Stravasi Iatrogeni di Soluti Citotossici o Iperosmolari. Trattamento*.

[7] Onesti MG, Latini C, Amauro R, Scuderi N (1993). Lo stravaso di farmaci antiblastici come trauma del dorso della mano: protocollo di trattamento. *Rivista Italiana di Chirurgia Plastica*.

[8] Forrest RD (1982). Early history of wound treatment. *Journal of the Royal Society of Medicine*.

[9] Edlich RF, Rodeheaver GT, Thacker JG (2010). Revolutionary advances in the management of traumatic wounds in the emergency department during the last 40 years: part I. *Journal of Emergency Medicine*.

